# Supplementary material for: OCT Angiography in Noninfectious Uveitis: A Description of Five Cases and Clinical Applications
Source: Diagnostics (Basel). 2023 Mar 30;13(7):1296. doi: 10.3390/diagnostics13071296 (PMC10092962; doi:10.3390/diagnostics13071296)
Supplement: Supplementary file 1 [file diagnostics-13-01296-s001.zip › diagnostics-2204641-supplementary.pdf]

Supplemental Figures legends

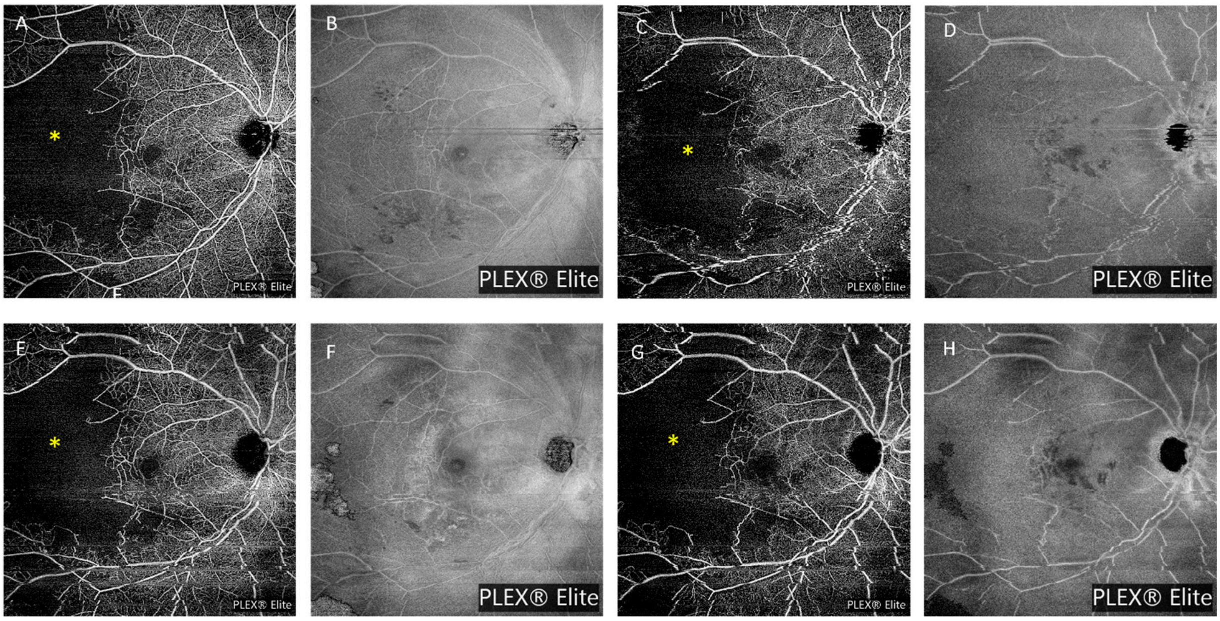

Supplemental Figure S1: Ischemic maculopathy A. Cirrus OCTA (12 x 12 mm) demonstrating an area of "flow deficit" or decreased signal in the superficial capillary plexus (SCP) corresponding to retinal ischemia in the temporal area; B. The border of nonperfusion is seen on the structural OCT in superficial capillary plexus; C. Cirrus OCTA (12 x 12 mm) demonstrating an area of "flow deficit" or decreased signal in the deep capillary plexus (SCP) corresponding to retinal ischemia in the temporal area; D. The border of nonperfusion is seen on the structural OCT in deep capillary plexus. E. Eleven-month follow-up OCTA that shows no change in flow deficit in superficial capillary plexus. F. Eleven-month follow-up, the border of nonperfusion is seen unchanged on the structural OCT in superficial capillary plexus. G. Eleven-month follow-up OCTA that shows no change in flow deficit in deep capillary plexus. H. Eleven-month follow-up, the border of nonperfusion is seen unchanged on the structural OCT in deep capillary plexus.

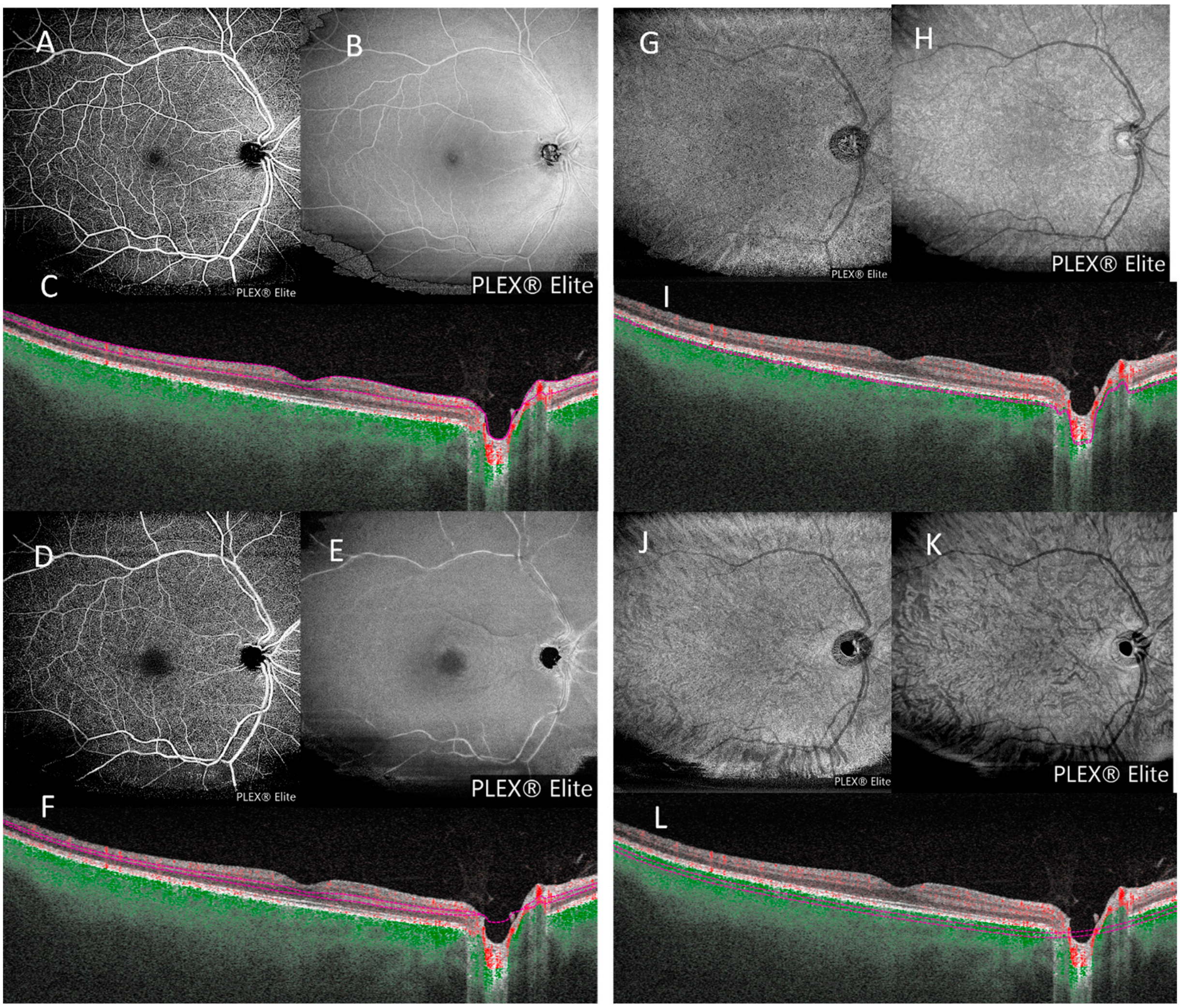

Supplemental Figure S2: Healthy control. Zeiss PLEX ® Elite 9000 OCTA (12x12 mm) of superficial capillary plexus (A), deep capillary plexus (D), choriocapillaris (G), choroid (J) and the corresponding inner en face structure (B, E, H, K) and corresponding Optical Coherence Tomography (OCT) B-scans (C, F, I, L).

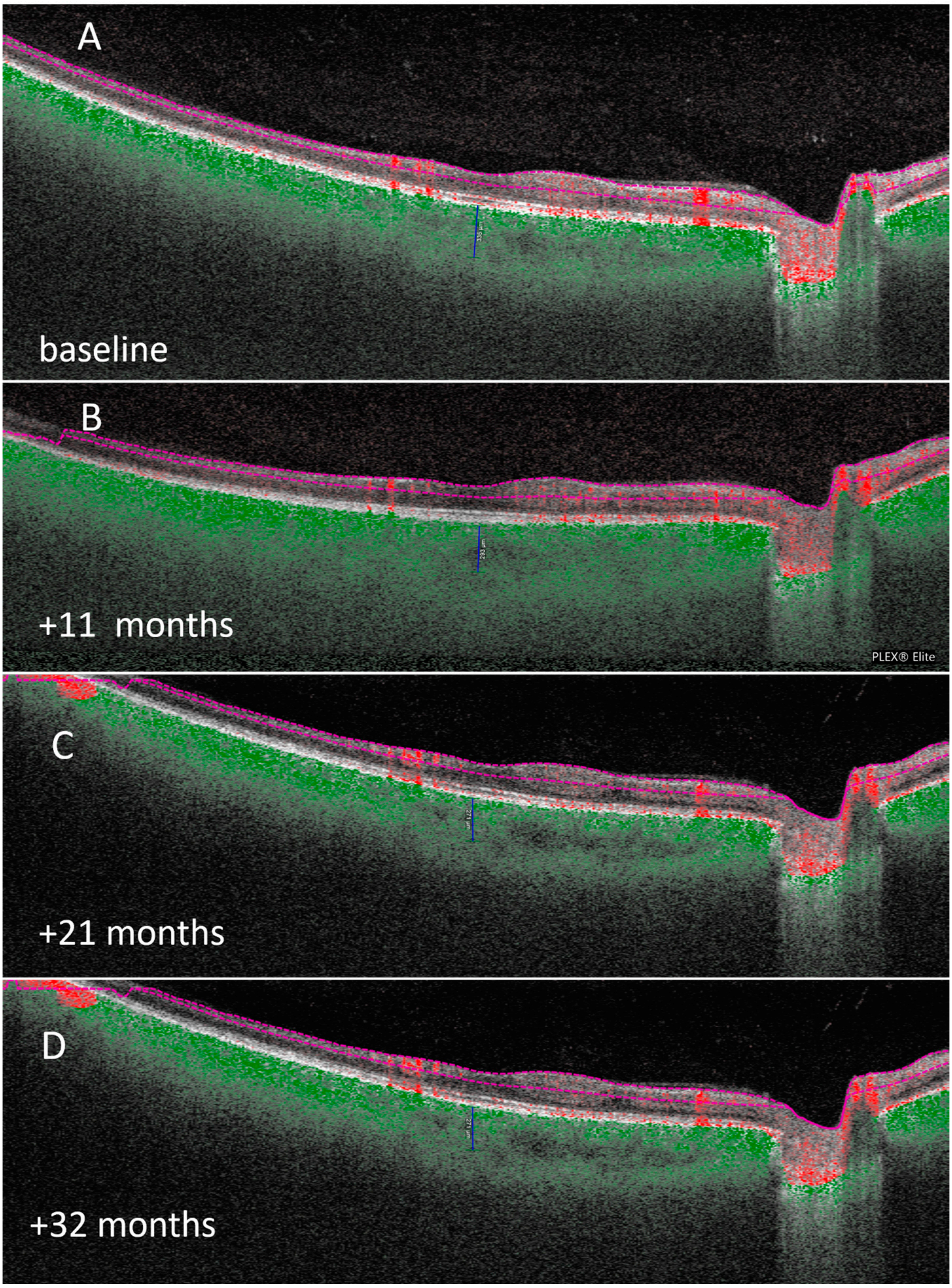

Supplemental Figure S3. Case 1. Ischemic retina in lupus retinopathy. A. The choroidal thickness measurements were obtained from manual segmentation of OCT B-scans at the fovea at baseline, +11 months, +21 months and +32 months.
